# COVID‐19 vaccines and risks of hematological abnormalities: Nested case–control and self‐controlled case series study

**DOI:** 10.1002/ajh.26478

**Published:** 2022-02-09

**Authors:** Chor‐Wing Sing, Casey Tze Lam Tang, Celine Sze Ling Chui, Min Fan, Francisco Tsz Tsun Lai, Xue Li, Eric Yuk Fai Wan, Carlos King Ho Wong, Esther Wai Yin Chan, Ivan Fan Ngai Hung, Anskar Yu‐Hung Leung, Ching‐Lung Cheung, Ian Chi Kei Wong

**Affiliations:** ^1^ Department of Pharmacology and Pharmacy, Li Ka Shing Faculty of Medicine The University of Hong Kong Hong Kong Special Administrative Region China; ^2^ Laboratory of Data Discovery for Health (D24H) Hong Kong Science Park Hong Kong Special Administrative Region China; ^3^ School of Nursing, Li Ka Shing Faculty of Medicine The University of Hong Kong Hong Kong Special Administrative Region China; ^4^ School of Public Health, Li Ka Shing Faculty of Medicine The University of Hong Kong Hong Kong Special Administrative Region China; ^5^ Centre for Safe Medication Practice and Research, Department of Pharmacology and Pharmacy, Li Ka Shing Faculty of Medicine The University of Hong Kong Hong Kong Special Administrative Region China; ^6^ Department of Medicine, Li Ka Shing Faculty of Medicine The University of Hong Kong Hong Kong Special Administrative Region China; ^7^ Department of Family Medicine and Primary Care, Li Ka Shing Faculty of Medicine The University of Hong Kong Hong Kong Special Administrative Region China; ^8^ Expert Committee on Clinical Events Assessment Following COVID‐19 Immunization, Department of Health The Government of the Hong Kong Special Administrative Region Hong Kong Special Administrative Region China; ^9^ Division of Haematology, Department of Medicine, Li Ka Shing Faculty of Medicine The University of Hong Kong Hong Kong Special Administrative Region China; ^10^ Research Department of Practice and Policy, School of Pharmacy University College London London UK

## Abstract

Several studies reported hematological abnormalities after vaccination against the coronavirus disease 2019 (COVID‐19). We evaluated the association between COVID‐19 vaccines (CoronaVac and BNT162b2) and hematological abnormalities. We conducted nested case–control and self‐controlled case series analyses using the data from the Hong Kong Hospital Authority and the Department of Health, HKSAR. Outcomes of interest were thrombocytopenia, leukopenia, and neutropenia. Adjusted odds ratios (aORs), incidence rate ratios (IRRs), and 95% confidence intervals (CIs) were estimated using conditional logistic regression. In total, 1 643 419 people received COVID‐19 vaccination (738 609 CoronaVac; 904 810 BNT162b2). We identified 457 and 422 cases after CoronaVac and BNT162b2 vaccination, respectively. For CoronaVac, the incidence of thrombocytopenia, leukopenia, and neutropenia was 2.51, 1.08, and 0.15 per 10 000 doses. For BNT162b2, the corresponding incidence was 1.39, 1.17, and 0.26 per 10 000 doses. The incidence per 10 000 COVID‐19 cases were 1254, 2341, and 884, respectively. We only observed an increased risk of leukopenia following the second dose of BNT162b2 (aOR 1.58, 95% CI 1.24–2.02; day 0–14, IRR 2.21; 95% CI 1.59–3.08). There was no increased risk of any hematological abnormalities after CoronaVac vaccination. We observed an increased risk of leukopenia shortly after the second dose of BNT162b2. However, the incidence was much lower than the incidence following severe acute respiratory syndrome coronavirus 2 (SARS‐CoV‐2) infections. There was no association between CoronaVac and hematological abnormalities. The benefits of vaccination against COVID‐19 still outweigh the risk of hematological abnormalities.

## 1 INTRODUCTION

The coronavirus disease 2019 (COVID‐19) pandemic has infected over 209 million people and caused more than 4.3 million deaths worldwide, as of August 2021.^1^ Vaccination is considered the most effective mean of controlling the pandemic. Traditional inactivated vaccines^2^, ^3^ and new viral vector–mRNA vaccines^4^, ^5^ have been developed with proven efficacy against COVID‐19 infections, hospitalization, and mortality.^6^, ^7^, ^8^ Many countries have authorized the emergency use of the vaccines and started a national program of COVID‐19 vaccination.

However, the safety of the COVID‐19 vaccines has been a major concern to the public, resulting in vaccine hesitancy, especially those developed by the new mRNA technology. Several case series reported hematological abnormalities, including thrombocytopenia and immune thrombocytopenic purpura (ITP) after vector‐based (e.g., ChAdOx1 nCoV‐19)^9^, ^10^ or mRNA‐based (e.g., BNT162b2) vaccination.^11^, ^12^ The phase 1 and 2 clinical trials of ChAdOx1 nCoV‐19 vaccine from AstraZeneca observed transient neutropenia in 46% of the recipients.^4^ These findings indicated that the vaccines may trigger the immune response affecting the blood system.

Given that hematological abnormalities may result in life‐threatening complications, estimating the risk of such serious adverse reactions is important. Currently, there is no real‐world study concerning the hematological effects in inactivated vaccines, for example, CoronaVac. Recent population‐based studies^13^, ^14^, ^15^ only investigated the risk of thrombocytopenia after COVID‐19 vaccines. However, whether the vaccines are associated with other hematological abnormalities such as decreased white blood cells (WBC) counts remains unknown. We conducted a population‐based study in Hong Kong to evaluate the association between hematological abnormalities (including thrombocytopenia, leukopenia, and neutropenia) and COVID‐19 vaccines (CoronaVac and BNT162b2), using nested case–control and self‐controlled case series (SCCS) analyses.

## 2 MATERIAL AND METHODS

### 2.1 Study design

We conducted nested case–control and SCCS analyses to evaluate the association between COVID‐19 vaccines and hematological abnormalities using patients in the Hong Kong Hospital Authority (HA). We also estimated the incidence of hematological abnormalities in people receiving COVID‐19 vaccination and people tested positive in the SARS‐CoV‐2 test (COVID‐19 cases), respectively. The mass COVID‐19 vaccination program in Hong Kong has broadly included people from different sectors, including healthy individuals (the rollout schedule is described in Table S1). Thus, people receiving vaccination may be healthier than those without receiving vaccination. Such concern has been acknowledged in the previous studies.^13^, ^14^ We, therefore, conducted SCCS analysis, a within‐individual comparison, to further minimize such selection bias and control unmeasured confounding, in addition to the nested case–control analysis.^16^ SCCS can be served as an internal validation for the results in the nested case–control analysis.

### 2.2 Data source

This study is part of the regulatory pharmacovigilance study initiated by the Department of Health (DH) of the Government of Hong Kong. Vaccination data were provided by the DH, which included all people receiving the COVID‐19 vaccine in Hong Kong. The Hong Kong government manages the allocation of vaccines to public and private sectors and tracks all individual vaccination records. Thus, data quality and representatives can be guaranteed. The data have been used to study the risk of Bell's palsy associated with COVID‐19 vaccines.^17^

Vaccination records were linked to the clinical data from the electronic healthcare database that is managed by the HA, a statutory body that provides subsidized public healthcare services and serves over 80% of the Hong Kong population.^18^ The database has been used to conduct the safety study of COVID‐19 vaccines^17^ and pharmacoepidemiological studies.^19^, ^20^, ^21^ In this study, we acquired anonymized patient‐level data between January 1, 2018 and July 31, 2021.

### 2.3 Study cohort

People aged ≥16 and who had ever used the HA service (inpatients, outpatients, or emergency) between January 1, 2018 and July 31, 2021 were included. We excluded those who had any medical conditions affecting the blood counts prior to the hematological abnormality. These conditions included (i) history of the same hematological abnormality; (ii) history of cancer; (iii) recent chemotherapy, radiotherapy, or use of drugs for malignant disease and immunosuppression within 90 days prior; and (iv) diseases affecting the blood cells (myelodysplastic syndromes, iron–vitamin B12 deficiency anemia, aplastic anemia, hypersplenism, chronic renal failure, viral infection, alcohol abuse–alcoholic liver diseases). We further excluded people with heparin use within 14 days prior to thrombocytopenia.^22^ All diagnoses were defined by the International Classification of Diseases, Ninth Revision codes, whereas prescriptions were defined by British national formulary (BNF) or local drug codes (Table S2).

### 2.4 Vaccination

The COVID‐19 Vaccination Program in Hong Kong provides free administration of CoronaVac and BNT162b2 since February 23, 2021 and March 6, 2021, respectively.^23^ The recommended schedule after the first dose is 28 days for CoronaVac and 21 days for BNT162b2. Recipients can schedule their second dose vaccination as long as it is not shorter than the recommended schedule. By July 2021, almost 60% of the Hong Kong population have received at least one dose of the vaccine.^23^

### 2.5 Outcome assessments

The outcomes of interest were thrombocytopenia, leukopenia, and neutropenia. To identify the events, we reviewed the laboratory records of platelet counts (<150 × 10^9^/L for thrombocytopenia^24^), total WBC counts (<4 × 10^9^/L for leukopenia^25^), and neutrophil counts (<1.5 × 10^9^/L for neutropenia^26^) in people admitted to hospitals between February 23, 2021 and July 31, 2021. Given that hematological abnormalities could be asymptomatic, we reviewed the laboratory records 7 days before–after admission to identify the onset time of the event. The index date was the date of the first laboratory record fulfilling the outcome definition.

### 2.6 Incidence of hematological abnormalities

Incidence was defined as new onset of hematological abnormality since 2018. Apart from the incidence per vaccine doses administered, we also estimated the incidence per COVID‐19 cases from January 23, 2020 (the first COVID‐19 case in Hong Kong) to July 31, 2021. Cases were defined as the onset of the event within 28 days following vaccination or positive result of the SARS‐CoV‐2 test. We used 28 days as the time period to define cases, which is in line with the published literatures.^13^, ^14^ Follow‐up was censored on 28 days, date of death, end of the study period, whichever occurred earlier. For the first dose, follow‐up was also censored on the date of the second dose vaccination. Severe thrombocytopenia and neutropenia may result in life‐threatening complications such as internal bleeding and serious infections. Therefore, we further estimated the incidence for mild, moderate, and severe cases, according to the corresponding blood counts (Platelet counts <100–150, 50–100, <50 × 10^9^/L, for thrombocytopenia^27^; Neutrophil counts <1 to <1.5, 0.5–1, <0.5 × 10^9^/L, for neutropenia^28^).

### 2.7 Nested case–control analysis

People who had the outcome of interest were considered cases. Controls were those who were admitted to hospitals during the study period and had not yet experienced the outcome of interest. The index date for control was defined as the admission date. Exclusion criteria for controls were the same as the study cohort aforementioned. To minimize selection bias from healthier vaccine recipients, we further excluded people who were ever admitted to hospitals in the past 3 years. We matched cases and controls by age, sex, index date (±7 days), and the Charlson's comorbidity index (0, 1–2, 3–4, ≥5) at a ratio of 1:10, using random sampling with replacement. Cases with less than 10 matched controls were excluded.

Exposure of interest was the receipt of either CoronaVac or BNT162b2 within 28 days^14^, ^15^ prior to the index date. Those who received the vaccine ≥28 days earlier than the index date were excluded. We evaluated the vaccine effect by dose. Cases that occurred within 28 days of the first dose and before the second dose were included for the analysis of first dose effect, whereas cases that occurred within 28 days of the second dose were included for the analysis of second dose effect.

### 2.8 SCCS analysis

In the study cohort, people who had the outcome of interest were included in the SCCS analysis. Observation period of an individual started from February 23, 2021 to July 31, 2021. For vaccinated people, the date of vaccination was considered day 0. A risk period was defined as the period from days 0–27 following the vaccination, which was stratified into days 0–13 and days 14–27. Control period was any other nonrisk periods within the observation period. Figure S1 illustrates the schema of SCCS design.

To ensure unbiased estimates, three assumptions should be met^16^: (1) events must be independently recurrent; (2) occurrence of an event should not affect subsequent exposure; and (3) events should not censor observation period. Thus, only first event was included because recurrent events could be dependent (violation of assumption 1). In addition, people with previous hematological abnormalities were unlikely to receive the vaccine (violation of assumption 2). Similarly, events that occurred after the first dose may decrease the probability of second dose vaccination. Thus, we applied the modified SCCS extension “eventdepenexp” in the R‐package “SCCS,” which is designed to fit the model with event‐dependent exposures.^29^ Cases without vaccination were included in the extension model for adjustment. Furthermore, we excluded people who died during the study (violation of assumption 3).

### 2.9 Statistical analysis

#### 2.9.1 Incidence rate of hematological abnormalities

The numerator was the total number of cases after the first or second dose. The denominator was the number of doses administered or COVID‐19 cases. Incidence rate (IR) and 95% confidence intervals (CIs) were estimated using Poisson regression.

#### 2.9.2 Nested case–control analysis

Conditional logistic regression stratified by match pairs was used. Standardized mean difference (SMD) of the baseline variables between matched cases and controls were calculated, and those with SMD <0.2 were adjusted in the model. The model was further adjusted for confounding variables,^30^, ^31^ which included the medical history of diabetes, hypertension, rheumatoid arthritis, systemic lupus erythematosus, psoriasis, thyroid disorders, moderate–server liver diseases; recent (90 days prior) prescription of lipid‐lowering agents, antiepileptic drugs, diuretics, oral anticoagulants, nonsteroidal anti‐inflammatory drugs, antithyroid drugs, antidepressants, antiplatelet drugs, and anti‐arrhythmia drugs.

#### 2.9.3 SCCS analysis

The IRs of hematological abnormalities between the risk and control periods were compared. Incidence rate ratios (IRRs) and 95% CIs were estimated using conditional logistic regression. Seasonal effect was adjusted for each month.

#### 2.9.4 Sample size calculation

Simpson et al.^14^ reported an adjusted relative risk of 2.8 for thrombocytopenia in the nested case–control analysis and an IRR of 1.98 for ITP in the SCCS analysis. Based on these estimates and the 60% of the vaccinated population in Hong Kong, 45 cases and 450 controls (1–10 match) are required in the nested case–control analysis, whereas 155 cases were required in the SCCS analysis to achieve 80% statistical power to detect association at 0.05 significance level.

#### 2.9.5 Additional analysis

Given that most thrombocytopenic events were reported among people aged <60,^9^, ^32^ we conducted subgroup analysis stratified by age <60 years and age ≥60 years. In addition, we conducted an additional analysis by excluding COVID‐19 cases. Furthermore, to investigate any delayed onset of hematological abnormality beyond 28 days, we used a longer time period of 84 days to define cases associated with the second dose vaccination, while the time period for the first dose vaccination remained unchanged because people have generally received the second dose 21 and 28 days after the first dose for BNT162b2 and CoronaVac, respectively, as recommended by the manufacturers.

The statistical analyses were done by two researchers (C.W.S and C.T.L.T) independently using R software version 3.6.1 and their results were cross‐checked. A two‐tailed *p*‐value <.05 was considered significant.

## 3 RESULTS

In total, we identified 3 983 529 people aged 16–120 years who used the HA service. Of these, 1 643 419 people received at least one dose of COVID‐19 vaccine (738 609 CoronaVac; 904 810 BNT162b2) and 74.2% of them received the second dose (75.1% CoronaVac; 73.4% BNT162b2). Compared to BNT162b2, the proportion of elderly in people receiving CoronaVac was higher (age ≥60: 31.3% in CoronaVac vs. 27.0% in BNT162b2), whereas the sex distribution was similar (men: 47.0% in CoronaVac vs. 45.5% in BNT162b2). In the nested case–control analysis, 7086 cases and 46 899 controls were matched. The characteristics of matched cases and controls are shown in Table 1. In SCCS analysis, 14 715 cases (11 540 unvaccinated and 3175 vaccinated) were included. The characteristics of these people are shown in Table 2. The screening flow chart is shown in Figure S2. For sensitivity analysis using a longer time period, given a maximum follow‐up period of 130 days for the second dose vaccination, almost 90% of hematological abnormality cases were captured within 84 days (Figure S3). Given the small number of cases beyond 28 days, we split the risk period beyond 28 days into two 4‐week periods, that is, day 28–55 and day 56–83 in the SCCS analysis.

**TABLE 1:** People characteristics in nested case–control analysis after matching

	Thrombocytopenia			Leukopenia			Neutropenia		
	Control	Case	SMD^a^	Control	Case	SMD	Control	Case	SMD
People, *n*	52 394	5538		18 310	1947		3284	349	
Mean age at onset (SD)	66 (19.7)	65 (19.5)	0.016	57 (19.3)	56 (19.0)	0.009	52 (19.8)	51 (19.7)	0.005
Male, *n* (%)	27 130 (51.8)	2911 (52.6)	0.016	6602 (36.1)	706 (36.3)	0.004	1085 (33.0)	118 (33.8)	0.016
Charlson's comorbidity index			0.010			0.010			0.004
0	36 251 (69.2)	3858 (69.7)		14 852 (81.1)	1587 (81.5)		0 (0.0)	0 (0.0)	
1–2	16 069 (30.7)	1672 (30.2)		3439 (18.8)	358 (18.4)		475 (14.5)	50 (14.3)	
3–4	74 (0.1)	8 (0.1)		19 (0.1)	2 (0.1)		0 (0.0)	0 (0.0)	
≥5	0 (0.0)	0 (0.0)		0 (0.0)	0 (0.0)		0 (0.0)	0 (0.0)	
Medical history									
Congestive heart failure	887 (1.7)	160 (2.9)	0.080	207 (1.1)	35 (1.8)	0.056	21 (0.6)	6 (1.7)	0.100
Hypertension	18 934 (36.1)	1590 (28.7)	0.159	4697 (25.7)	354 (18.2)	0.181	706 (21.5)	43 (12.3)	0.247
Vascular disease	3536 (6.7)	398 (7.2)	0.017	793 (4.3)	72 (3.7)	0.032	117 (3.6)	7 (2.0)	0.095
Ischemic stroke	1933 (3.7)	177 (3.2)	0.027	394 (2.2)	21 (1.1)	0.085	54 (1.6)	4 (1.1)	0.042
Diabetes	10 856 (20.7)	981 (17.7)	0.076	2284 (12.5)	197 (10.1)	0.074	349 (10.6)	28 (8.0)	0.090
COPD	1513 (2.9)	97 (1.8)	0.076	359 (2.0)	38 (2.0)	0.001	34 (1.0)	5 (1.4)	0.036
Moderate–severe liver disease	0 (0.0)	4 (0.1)	0.038	0 (0.0)	0 (0.0)	<0.001	0 (0.0)	0 (0.0)	<0.001
Rheumatoid arthritis and SLE	135 (0.3)	12 (0.2)	0.008	42 (0.2)	21 (1.1)	0.106	5 (0.2)	5 (1.4)	0.145
Hyper−hypo‐thyroidism	911 (1.7)	121 (2.2)	0.032	370 (2.0)	74 (3.8)	0.106	54 (1.6)	29 (8.3)	0.310
Recent prescription (90 days prior)									
Lipid‐lowering agents	14 389 (27.5)	1260 (22.8)	0.109	3231 (17.6)	251 (12.9)	0.132	461 (14.0)	27 (7.7)	0.203
Anti‐arrhythmic drugs	77 (0.1)	58 (1.0)	0.117	19 (0.1)	5 (0.3)	0.036	4 (0.1)	1 (0.3)	0.037
Oral anticoagulants	717 (1.4)	82 (1.5)	0.009	220 (1.2)	15 (0.8)	0.044	29 (0.9)	7 (2.0)	0.094
Antiplatelets	7272 (13.9)	756 (13.7)	0.007	1495 (8.2)	157 (8.1)	0.004	216 (6.6)	21 (6.0)	0.023
Antidepressants	2419 (4.6)	208 (3.8)	0.043	842 (4.6)	74 (3.8)	0.040	147 (4.5)	19 (5.4)	0.045
NSAIDs	3322 (6.3)	326 (5.9)	0.019	1303 (7.1)	153 (7.9)	0.028	228 (6.9)	38 (10.9)	0.139
Antiepileptic drugs	1028 (2.0)	200 (3.6)	0.100	363 (2.0)	42 (2.2)	0.012	88 (2.7)	4 (1.1)	0.112
Antithyroid drugs	188 (0.4)	17 (0.3)	0.009	70 (0.4)	15 (0.8)	0.051	15 (0.5)	8 (2.3)	0.158

Abbreviations: COPD, chronic obstructive pulmonary disease; NSAID, nonsteroidal anti‐inflammatory drugs; SLE, systemic lupus erythematosus; SMD standardized mean difference.

^a^ Variables with SMD <0.2 were further adjusted in the model.

**TABLE 2:** People characteristics in self‐controlled case series analysis

Thrombocytopenia	Unvaccinated	CoronaVac	BNT162b2
People, *n*	8571	1166	975
Mean age at onset (SD)	69 (19.7)	61 (15.4)	52 (17.9)
Aged <60, *n* (%)	2237 (26.1)	508 (43.6)	614 (63)
Male, *n* (%)	4116 (48)	715 (61.3)	549 (56.3)
Leukopenia			
People, *n*	3396	550	655
Mean age at onset (SD)	66 (19.4)	57 (14.6)	47 (16.7)
Aged <60, *n* (%)	1168 (34.4)	313 (56.9)	482 (73.6)
Male, *n* (%)	1400 (41.2)	220 (40)	252 (38.5)
Neutropenia			
People, *n*	686	95	160
Mean age at onset (SD)	62 (20.8)	54 (15.3)	43 (16.5)
Aged <60, *n* (%)	283 (41.3)	59 (62.1)	128 (80)
Male, *n* (%)	262 (38.2)	40 (42.1)	48 (30)

### 3.1 Incidence of hematological abnormalities

Among CoronaVac recipients, we identified 219 cases (146 thrombocytopenia, 64 leukopenia, and 9 neutropenia) within 28 days following the first dose and 238 cases (160 thrombocytopenia, 68 leukopenia, and 10 neutropenia) within 28 days following the second dose. In total, the incidence per 10 000 CoronaVac doses for thrombocytopenia, leukopenia, and neutropenia were 2.51 (95% CI 2.24–2.81), 1.08 (95% CI 0.90–1.28), and 0.15 (95% CI 0.09–0.24), respectively. The incidence of thrombocytopenia and neutropenia stratified by severity are shown in Table S3.

Among BNT162b2 recipients, we identified 151 cases (82 thrombocytopenia, 60 leukopenia, and 9 neutropenia) within 28 days following the first dose and 271 cases (126 thrombocytopenia, 114 leukopenia, and 31 neutropenia) within 28 days following the second dose. The incidence per 10 000 BNT162b2 doses for thrombocytopenia, leukopenia, and neutropenia were 1.39 (95% CI 1.21–1.59), 1.17 (95% CI 1–1.35), and 0.26 (95% CI 0.19–0.36), respectively. The incidence of thrombocytopenia and neutropenia stratified by severity were shown in Table S3.

We identified 11 277 COVID‐19 cases. Of these, we observed 5053 cases (1415 thrombocytopenia, 2641 leukopenia, and 997 neutropenia) within 28 days following the test. The incidence of thrombocytopenia, leukopenia, and neutropenia per 10 000 COVID‐19 cases were 1254 (95% CI 1190–1322), 2341 (95% CI 2253–2433), and 884 (95% CI 830–941), respectively.

### 3.2 Risk of thrombocytopenia

We included 5538 thrombocytopenia cases in the nested case–control and 10 712 cases (8571 unvaccinated, 1166 CoronaVac, 975 BNT162b2) in the SCCS analysis. We did not observe any increased risk of thrombocytopenia after CoronaVac in neither the nested case–control (Table 3) nor SCCS analysis (Table 4). There was a lower risk after the first dose of BNT162b2 in nested case–control (aOR 0.75; 95% CI 0.62–0.92, Table 3) but not in SCCS analysis (Table 4). Similar findings were shown in the subgroup (Tables S4–S7) and additional analyses excluding COVD‐19 cases (Tables S8 and S9). In the sensitivity analysis examining the association beyond 28 days, no increased risk was observed in the nested case–control and SCCS analysis for both vaccines (Tables S10 and S11).

**TABLE 3:** Association between COVID‐19 vaccines and hematological abnormalities in nested case–control analysis

Exposure	Case	Control	Odds ratio (OR) (95% CI)	Adjusted^a^ OR (95% CI)
Thrombocytopenia				
Events after first dose and before second dose				
Not vaccinated	5059	45 924	1	1
CoronaVac	166	1377	1.05 (0.89–1.24)	1.07 (0.90–1.26)
BNT162b2	107	1203	0.76 (0.62–0.93)	0.75 (0.62–0.92)
Events after second dose				
Not vaccinated	5059	45 335	1	1
CoronaVac	107	1019	0.89 (0.72–1.09)	0.88 (0.72–1.08)
BNT162b2	99	967	0.86 (0.70–1.06)	0.85 (0.69–1.05)
Leukopenia				
Events after first dose and before second dose				
Not vaccinated	1689	15 124	1	1
CoronaVac	68	579	1.02 (0.79–1.32)	1.01 (0.78–1.31)
BNT162b2	65	575	0.96 (0.74–1.25)	0.96 (0.74–1.25)
Events after second dose				
Not vaccinated	1689	15 067	1	1
CoronaVac	42	408	0.89 (0.64–1.22)	0.88 (0.64–1.22)
BNT162b2	83	459	1.56 (1.22–1.98)	1.58 (1.24–2.02)
Neutropenia				
Events after first dose and before second dose				
Not vaccinated	297	2678	1	1
CoronaVac	9	99	0.80 (0.40–1.60)	0.73 (0.35–1.49)
BNT162b2	16	123	1.11 (0.64–1.92)	1.13 (0.65–1.98)
Events after second dose				
Not vaccinated	297	2702	1	1
CoronaVac	6	65	0.81 (0.35–1.88)	0.83 (0.35–1.94)
BNT162b2	21	68	2.63 (1.58–4.38)	2.74 (1.63–4.61)

^a^ Model adjusted for medical history of diabetes, hypertension, rheumatoid arthritis, systemic lupus erythematosus, psoriasis, thyroid disorders, moderate–server liver diseases; recent (90 days prior) prescription of lipid‐lowering agents, antiepileptic drugs, diuretics, oral anticoagulants, nonsteroidal anti‐inflammatory drugs, antithyroid drugs, antipsychotic drugs, antiplatelet drugs, anti‐arrhythmic drugs.

**TABLE 4:** Association between COVID‐19 vaccines and hematological abnormalities in SCCS analysis

Risk period	Event	Person‐years	IR	IRR^a^ (95% CI)
Thrombocytopenia				
CoronaVac				
Control period	9371	4109.18	2.28	
1st dose, day 0–13	103	39.38	2.62	1.04 (0.80–1.34)
1st dose, day 14–27	116	37.70	3.08	1.18 (0.93–1.50)
2nd dose, day 0–13	80	27.49	2.91	1.08 (0.80–1.46)
2nd dose, day 14–27	67	24.94	2.69	0.92 (0.68–1.25)
BNT162b2				
Control period	9287	4060.01	2.29	
1st dose, day 0–13	80	32.74	2.44	0.96 (0.72–1.28)
1st dose, day 14–27	59	21.62	2.73	1.00 (0.74–1.35)
2nd dose, day 0–13	75	22.07	3.40	1.19 (0.89–1.59)
2nd dose, day 14–27	45	19.11	2.35	0.79 (0.56–1.12)
Leukopenia				
CoronaVac				
Control period	3787	1658.28	2.28	
1st dose, day 0–13	50	18.49	2.70	1.01 (0.69–1.47)
1st dose, day 14–27	46	17.26	2.67	0.90 (0.61–1.31)
2nd dose, day 0–13	33	12.42	2.66	0.95 (0.61–1.46)
2nd dose, day 14–27	30	11.33	2.65	0.90 (0.58–1.39)
BNT162b2				
Control period	3838	1698.75	2.26	
1st dose, day 0–13	64	21.75	2.94	1.22 (0.87–1.72)
1st dose, day 14–27	39	14.64	2.66	1.05 (0.72–1.52)
2nd dose, day 0–13	81	15.08	5.37	2.21 (1.59–3.08)
2nd dose, day 14–27	29	13.25	2.19	0.91 (0.58–1.42)
Neutropenia				
CoronaVac				
Control period	756	329.56	2.29	
1st dose, day 0–13	12	3.19	3.76	1.31 (0.59–2.89)
1st dose, day 14–27	4	3.07	1.30	0.50 (0.18–1.37)
2nd dose, day 0–13	5	2.17	2.30	0.92 (0.33–2.52)
2nd dose, day 14–27	4	1.99	2.01	0.69 (0.22–2.15)
BNT162b2				
Control period	801	352.16	2.27	
1st dose, day 0–13	12	5.47	2.19	0.41 (0.15–1.09)
1st dose, day 14–27	6	3.55	1.69	0.28 (0.10–0.85)
2nd dose, day 0–13	19	3.81	4.99	1.10 (0.52–2.31)
2nd dose, day 14–27	8	3.29	2.43	0.60 (0.25–1.46)

Abbreviations: IR, incidence rate; IRR, incidence rate ratio; SCCS, self‐controlled case series.

^a^ IRR was estimated using modified SCCS extension “eventdepenexp” model.

### 3.3 Risk of leukopenia

In total, 1947 leukopenia cases were included in the nested case–control and 4601 cases (3396 unvaccinated, 550 CoronaVac, 655 BNT162b2) in the SCCS analysis. There was no increased risk of leukopenia following CoronaVac vaccination in neither the nested case–control (Table 3) nor SCCS analysis (Table 4). In contrast, we observed an increased risk of leukopenia following the second dose of BNT162b2 in the nested case–control analysis (aOR 1.58; 95% CI 1.24–2.02, Table 3). In the SCCS analysis, we further found that the increased risk was only significant within the first 2 weeks following the second dose (IRR 2.21; 95% CI 1.59–3.08, Table 4). The association remained significant after excluding COVID‐19 cases (Tables S8 and S9). In subgroup analysis, we only observed similar results in people aged <60 years but not in people aged ≥60 years (Tables S4–S7). For the association beyond 28 days of vaccination, the findings in the nested case–control analysis were similar to the main analysis (Table S10). In the SCCS analysis, there was an increased risk on day 28–55 post second dose CoronaVac (IRR 2.15, 95% CI 1.25–3.71, Table S11).

### 3.4 Risk of neutropenia

We included 349 neutropenia cases in the nested case–control and 941 cases (686 unvaccinated, 95 CoronaVac, 160 BNT162b2) in the SCCS analysis. We did not observe any increased risk of neutropenia following CoronaVac vaccination in neither nested case–control (Table 3) nor SCCS analysis (Table 4). There was an increased risk after second dose of BNT162b2 in nested case–control analysis (aOR 2.74, 95% CI 1.63–4.61, Table 3). However, such association was not observed in the SCCS analysis (Table 4). On the other hand, there was no association after the first dose of BNT162b2 in the nested case–control analysis, but a lower risk was observed in the SCCS analysis (day14‐27, IRR 0.28, 95% CI 0.10–0.85, Table 4). Similar results were obtained in the additional analysis excluding COVID‐19 cases and using a longer time period of 84 days (Tables S8–S11). In subgroup analysis, an increased risk was observed only in people aged <60 in the nested case–control analysis (Tables S4–S7).

## 4 DISCUSSION

This real‐world study showed an increased risk of leukopenia following the second dose of BNT162b2 as shown in both nested case–control and SCCS analyses. We observed an increased risk of neutropenia following the second dose of BNT162b2 in the nested case–control but not in the SCCS analysis. No association between BNT162b2 and thrombocytopenia was identified. Similarly, there was no association between CoronaVac and any hematological abnormalities. To the best of our knowledge, this is the first real‐world study reporting the safety of CoronaVac regarding the hematological abnormalities.

We reported an increased risk of leukopenia among people receiving BNT162b2 vaccine for the first time. Leukopenia was not previously reported in the clinical trial of BNT162b2 vaccine,^5^ which is likely due to the limited sample size in the trial to detect rare adverse events. There is no other published population‐based study investigating changes in WBC counts after COVID‐19 vaccination.

Previous studies showed that the risk of thrombocytopenia varied across COVID‐19 vaccines. Pottegård et al.^13^ conducted a population‐based cohort study in Denmark and Norway. They found that people receiving ChAdOx1‐S/nCoV‐19 vaccine had an increased risk of thrombocytopenia with a standardized morbidity ratio of 3.02 (95% CI 1.76–4.83), compared to the general population. Similarly, Simpson et al.^14^ reported an increased risk of ITP among 1.7 million people receiving ChAdOx1‐S/nCoV‐19 vaccine in Scotland in both nested case–control (aRR 5.77, 95% CI, 2.41–13.83) and SCCS analysis (IRR 1.98, 95% CI, 1.29–3.02). A study in UK by Hippisley‐Cox et al.^15^ also supported the association. However, both Simpson et al. and Hippisley‐Cox et al. did not observe any association between BNT162b2 vaccine and thrombocytopenia/ITP. These findings suggested that the risk of thrombocytopenia seems to be elevated in vector‐based vaccines (e.g., ChAdOx1‐S/nCoV‐19), but not mRNA‐based (e.g., BNT162b2) vaccines. Our study further showed that such association was not observed in inactivated vaccine (CoronaVac) either. Notably, previous studies conducted by Simpson et al. and Hippisley‐Cox et al. only studied the first dose of the vaccine due to the limited sample size in people receiving the second dose. Our study further provided evidence of no association with thrombocytopenia in the second dose. This finding was in line with the recently published surveillance after COVID‐19 mRNA vaccination.^33^

Most findings were consistent in the nested case–control and SCCS analyses. However, we observed an increased risk of neutropenia following BNT162b2 vaccination in the nested case–control but not in the SCCS analysis. Indeed, nested case–control analysis could have residual confounding that might bias the estimates. Therefore, SCCS analysis was conducted to account for such residual confounding, and the results in the SCCS did not support the association between risk of neutropenia and BNT162b2. Further investigation is warranted to validate the findings.

The mechanism of leukopenia after BNT162b2 vaccination is unclear. However, decreased WBC counts after influenza vaccination was reported in early case reports,^34^, ^35^ suggesting that the adverse events could be due to a general immune response rather than a vaccine‐specific effect. In agreement with this hypothesis, the association was only observed in the first 2 weeks of the second dose but not in the first dose in our study. Indeed, a recent study in Hong Kong by Lim et al.^36^ showed that the antibody concentrations after the second dose of BNT162b2 increased substantially, compared with the antibody concentrations after the first dose. This indicated that the immune response after the second dose is stronger than the first dose, which matches our findings. The increased risk observed in people aged <60 years but not in people aged ≥60 years in our study further supports the hypothesis for the mechanism as young adults generally have stronger immune responses compared with the elderly. Conversely, the immunogenicity of CoronaVac, as reported in Lim et al., was much lower than that of BNT162b2. This could potentially explain the null association in CoronaVac.

Neutrophils are the most abundant WBC. In this study, we only observed a significant decrease in total WBC counts but not neutrophil counts. This observation was similar to an early study by Cummins et al., which observed a significant decline in total WBC and lymphocytes counts, but not in neutrophil counts after influenza vaccination.^34^ In the early phase study of the mRNA vaccine, decreases in lymphocyte counts were observed in another vaccine candidate BNT162b1.^37^ Thus, it is possible that the leukopenia after BNT162b2 was driven by lymphocytes rather than neutrophils. Since lymphocytes counts and other WBC counts data were not available, we cannot investigate this hypothesis.

Low platelets–WBC counts could result in serious complications such as internal bleeding and severe infections. However, our study showed that the incidence of hematological abnormalities after COVID‐19 vaccination was rare with only 0.2–2.5 cases per 10 000 vaccine doses, which was much lower than that in COVID‐19 cases (884–2341 per 10 000 cases). In addition, a majority of thrombocytopenia (88.7%) and neutropenia (76.3%) were mild cases. These indicated that the risk and severity of hematological abnormalities following COVID‐19 vaccination was minimal compared to COVID‐19 infection.

In the sensitivity analysis, we further evaluated the association beyond 28 days of vaccination. In SCCS analysis, an increased risk of leukopenia was shown in day 28–55 post second dose CoronaVac but not in the first 28 days. Such association was not reported in the nested case–control analysis. Given the inconsistent findings in the SCCS and nested case–control analysis, we cannot draw a conclusion on the association 28–55 days post second dose. However, we cannot rule out a possible late‐onset of leukopenia after CoronaVac vaccination, which might be due to the suppression of bone marrow resulting in the reduced production of WBC. Such adverse effect has been reported for some medications such as anticonvulsants^38^ and antipsychotics^39^ but rarely for vaccines. Thus, further studies are warranted to validate the findings. In addition, the infection rate that required hospitalization and multiple antibiotics was only 4.8% (data not shown) out of these delayed leukopenia cases in CoronaVac group, showing limited clinical significance even if the association is valid.

Our study has several strengths. First, it provided real‐world evidence on the safety of COVID‐19 vaccination, especially the inactivated vaccine CoronaVac. Second, we used laboratory values to ascertain the hematological abnormalities, which are more accurate than using diagnosis codes. Third, most findings in nested case–control and SCCS analysis were consistent, which could serve as an internal validation of the results. However, there were limitations in the study. First, we cannot exclude some ethnic groups, which are more common to have benign neutropenia^40^ due to the unknown information on ethnicity. However, we believed that this limitation unlikely impacted the findings, given that ethnic minorities such as Indonesians and Filipinos constitute only 8% of the total population in Hong Kong.^41^ Second, the predominantly Chinese population in the study cohort may limit the findings' generalizability to other populations. Thus, replication of the study in other populations is warranted. Third, the study was limited to vaccine recipients who used HA service since 2018. Thus, the effects of the vaccines on healthy individuals may not be captured. Fourth, we did not have differential WBC counts other than neutrophils to investigate, which cell types contributed to the leukopenia.

## 5 CONCLUSION

We observed an increased risk of leukopenia shortly after the second dose of BNT162b2, but the incidence was much lower than in people with SARS‐CoV‐2 infection. We did not observe any risk of hematological abnormalities following CoronaVac vaccination. The findings suggested that the beneficial effects of COVID‐19 vaccines, CoronaVac and BNT162b2, still outweigh the risk of hematological abnormalities.

## CONFLICT OF INTEREST

Chor‐Wing Sing has received grants from the Hong Kong Health and Medical Research Fund (HMRF, HKSARS) outside the submitted work. Celine Sze Ling Chui has received grants from the Food and Health Bureau of the Hong Kong Government, Hong Kong Research Grant Council (RGC), Hong Kong Innovation and Technology Commission, Pfizer, IQVIA, and Amgen; personal fees from Primevigilance Ltd.; outside the submitted work. Francisco Tsz Tsun Lai has been supported by the RGC Postdoctoral Fellowship under the Hong Kong RGC and has received research grants from Food and Health Bureau of the Government of the Hong Kong SAR, outside the submitted work. Xue Li received research grants from Hong Kong HMRF, RGC Early Career Scheme (RGC/ECS, HKSAR), Janssen, and Pfizer; internal funding from the University of Hong Kong; consultancy fee from Merck Sharp & Dohme, unrelated to this work. Eric Yuk Fai Wan has received research grants from the Food and Health Bureau of the Government of the Hong Kong SAR, and the Hong Kong RGC, outside the submitted work. Esther Wai Yin Chan reports honorarium from Hospital Authority, grants from Hong Kong RGC, grants from Research Fund Secretariat of the Food and Health Bureau, National Natural Science Fund of China, Wellcome Trust, Bayer, Bristol‐Myers Squibb, Pfizer, Janssen, Amgen, Takeda, Narcotics Division of the Security Bureau of HKSAR, outside the submitted work. Anskar Yu‐Hung Leung has received grants from Hong Kong RGC, HMRF, Innovation and Technology Commission of HKSAR. Ivan Fan Ngai Hung has received grants from Hong Kong RGC, HMRF, and Research Fund for the Control of Infectious Disease. Ching‐Lung Cheung has received grants from Hong Kong RGC, HMRF, the Narcotics Division of the Security Bureau of HKSAR, Amgen, outside the submitted work. Ian Chi Kei Wong reports research funding outside the submitted work from Amgen, Bristol‐Myers Squibb, Pfizer, Janssen, Bayer, GSK, Novartis, the Hong Kong RGC, and HMRF, National Institute for Health Research in England, European Commission, National Health and Medical Research Council in Australia, and also received speaker fees from Janssen and Medice in the previous 3 years. He is also an independent nonexecutive director of Jacobson Medical in Hong Kong.

## AUTHOR CONTRIBUTIONS

Chor‐Wing Sing designed the study, performed data analysis, and wrote the manuscript. Casey Tze Lam Tang performed data analysis. Min Fan assisted in the study design and data analysis. Celine Sze Ling Chui, Francisco Tsz Tsun Lai, Xue Li, Eric Yuk Fai Wan, Carlos King Ho Wong, Esther Wai Yin Chan, Ivan Fan Ngai Hung, and Anskar Yu‐Hung Leung critically reviewed and commented on the results, and manuscript. Ching‐Lung Cheung and Ian Chi Kei Wong designed the study, critically reviewed and commented on the results, and manuscript.

## Supporting information


**Table S1.** Rollout schedule for the COVID‐19 Vaccination Programme in Hong Kong.
**Table S2**. Definition for diagnosis, procedure and prescription.
**Table S3**. Incidence of thrombocytopenia and neutropenia stratified by disease severity after COVID‐19 vaccine.
**Table S4**. Subgroup analysis in people aged <60 in nested case‐control analysis.
**Table S5**. Subgroup analysis in people aged ≥60 in nested case‐control analysis.
**Table S6**. Subgroup analysis in people aged <60 in SCCS analysis.
**Table S7**. Subgroup analysis in people aged ≥60 in SCCS analysis.
**Table S8**. Sensitivity analysis excluding people with SARS‐CoV‐2 positive test in nested case‐control analysis.
**Table S9**. Sensitivity analysis excluding people with SARS‐CoV‐2 positive test in SCCS analysis.
**Table S10**. Sensitivity analysis using a longer timeframe (84 days) to define hematological abnormalities associated with COVID‐19 vaccination in nested case‐control analysis.
**Table S11**. Sensitivity analysis using a longer timeframe (84 days) to define hematological abnormalities associated with COVID‐19 vaccination in SCCS analysis.
**Figure S1**. Schema for SCCS design.
**Figure S2**. Screening flow chart for nested case‐control and SCCS analysis.
**Figure S3**. Time between second dose vaccination and outcome occurrence.

## Data Availability

Data will not be available for others as the data custodians have not given permission.

## References

[1] Dong E , Du H , Gardner L . An interactive web‐based dashboard to track COVID‐19 in real time. Lancet Infect Dis. 2020;20(5):533‐534. 32087114 10.1016/S1473-3099(20)30120-1 PMC7159018

[2] Zhang Y , Zeng G , Pan H , et al. Safety, tolerability, and immunogenicity of an inactivated SARS‐CoV‐2 vaccine in healthy adults aged 18‐59 years: a randomised, double‐blind, placebo‐controlled, phase 1/2 clinical trial. Lancet Infect Dis. 2021;21(2):181‐192. 33217362 10.1016/S1473-3099(20)30843-4 PMC7832443

[3] Xia S , Zhang Y , Wang Y , et al. Safety and immunogenicity of an inactivated SARS‐CoV‐2 vaccine, BBIBP‐CorV: a randomised, double‐blind, placebo‐controlled, phase 1/2 trial. Lancet Infect Dis. 2021;21(1):39‐51. 33069281 10.1016/S1473-3099(20)30831-8 PMC7561304

[4] Folegatti PM , Ewer KJ , Aley PK , et al. Safety and immunogenicity of the ChAdOx1 nCoV‐19 vaccine against SARS‐CoV‐2: a preliminary report of a phase 1/2, single‐blind, randomised controlled trial. Lancet. 2020;396(10249):467‐478. 32702298 10.1016/S0140-6736(20)31604-4 PMC7445431

[5] Polack FP , Thomas SJ , Kitchin N , et al. Safety and efficacy of the BNT162b2 mRNA Covid‐19 vaccine. N Engl J Med. 2020;383(27):2603‐2615. 33301246 10.1056/NEJMoa2034577 PMC7745181

[6] Lopez Bernal J , Andrews N , Gower C , et al. Effectiveness of the Pfizer‐BioNTech and Oxford‐AstraZeneca vaccines on covid‐19 related symptoms, hospital admissions, and mortality in older adults in England: test negative case‐control study. BMJ. 2021;373:n1088. 33985964 10.1136/bmj.n1088 PMC8116636

[7] Sadoff J , Gray G , Vandebosch A , et al. Safety and efficacy of single‐dose Ad26.COV2.S vaccine against Covid‐19. N Engl J Med. 2021;384(23):2187‐2201. 33882225 10.1056/NEJMoa2101544 PMC8220996

[8] Haas EJ , Angulo FJ , McLaughlin JM , et al. Impact and effectiveness of mRNA BNT162b2 vaccine against SARS‐CoV‐2 infections and COVID‐19 cases, hospitalisations, and deaths following a nationwide vaccination campaign in Israel: an observational study using national surveillance data. Lancet. 2021;397(10287):1819‐1829. 33964222 10.1016/S0140-6736(21)00947-8 PMC8099315

[9] Greinacher A , Thiele T , Warkentin TE , Weisser K , Kyrle PA , Eichinger S . Thrombotic thrombocytopenia after ChAdOx1 nCov‐19 vaccination. N Engl J Med. 2021;384(22):2092‐2101. 33835769 10.1056/NEJMoa2104840 PMC8095372

[10] Scully M , Singh D , Lown R , et al. Pathologic antibodies to platelet factor 4 after ChAdOx1 nCoV‐19 vaccination. N Engl J Med. 2021;384(23):2202‐2211. 33861525 10.1056/NEJMoa2105385 PMC8112532

[11] Tarawneh O , Tarawneh H . Immune thrombocytopenia in a 22‐year‐old post Covid‐19 vaccine. Am J Hematol. 2021;96(5):E133‐E134. 33476455 10.1002/ajh.26106 PMC8014773

[12] Lee EJ , Cines DB , Gernsheimer T , et al. Thrombocytopenia following Pfizer and Moderna SARS‐CoV‐2 vaccination. Am J Hematol. 2021;96(5):534‐537. 33606296 10.1002/ajh.26132 PMC8014568

[13] Pottegard A , Lund LC , Karlstad O , et al. Arterial events, venous thromboembolism, thrombocytopenia, and bleeding after vaccination with Oxford‐AstraZeneca ChAdOx1‐S in Denmark and Norway: population based cohort study. BMJ. 2021;373:n1114. 33952445 10.1136/bmj.n1114 PMC8097496

[14] Simpson CR , Shi T , Vasileiou E , et al. First‐dose ChAdOx1 and BNT162b2 COVID‐19 vaccines and thrombocytopenic, thromboembolic and hemorrhagic events in Scotland. Nat Med. 2021;27(7):1290‐1297. 34108714 10.1038/s41591-021-01408-4 PMC8282499

[15] Hippisley‐Cox J , Patone M , Mei XW , et al. Risk of thrombocytopenia and thromboembolism after covid‐19 vaccination and SARS‐CoV‐2 positive testing: self‐controlled case series study. BMJ. 2021;374:n1931. 34446426 10.1136/bmj.n1931 PMC8388189

[16] Weldeselassie YG , Whitaker HJ , Farrington CP . Use of the self‐controlled case‐series method in vaccine safety studies: review and recommendations for best practice. Epidemiol Infect. 2011;139(12):1805‐1817. 21849099 10.1017/S0950268811001531

[17] Wan EYF , Chui CSL , Lai FTT , et al. Bell's palsy following vaccination with mRNA (BNT162b2) and inactivated (CoronaVac) SARS‐CoV‐2 vaccines: a case series and nested case‐control study. Lancet Infect Dis. 2021;22:64‐72. 34411532 10.1016/S1473-3099(21)00451-5 PMC8367195

[18] Hospital Authority . Hospital Authority Statistical Report 2016–2017. 2018. Accessed August 8, 2019. http://www.ha.org.hk/haho/ho/stat/HASR16_17_2.pdf

[19] Lau WC , Chan EW , Cheung CL , et al. Association between dabigatran vs warfarin and risk of osteoporotic fractures among patients with nonvalvular atrial fibrillation. JAMA. 2017;317(11):1151‐1158. 28324091 10.1001/jama.2017.1363

[20] Wong AY , Root A , Douglas IJ , et al. Cardiovascular outcomes associated with use of clarithromycin: population based study. BMJ. 2016;352:h6926. 26768836 10.1136/bmj.h6926

[21] Cheung CL , Sing CW , Lau WCY , et al. Treatment with direct oral anticoagulants or warfarin and the risk for incident diabetes among patients with atrial fibrillation: a population‐based cohort study. Cardiovasc Diabetol. 2021;20(1):71. 33766030 10.1186/s12933-021-01263-0 PMC7993481

[22] Warkentin TE , Greinacher A . Heparin‐induced thrombocytopenia: recognition, treatment, and prevention: the seventh ACCP conference on antithrombotic and thrombolytic therapy. Chest. 2004;126(3 Suppl):311S‐337S. 15383477 10.1378/chest.126.3_suppl.311S

[23] The Government of the Hong Kong Special Administrative Region. COVID‐19 Vaccination Programme. 2021. Accessed August 19, 2021. https://www.covidvaccine.gov.hk/en/

[24] Guan WJ , Ni ZY , Hu Y , et al. Clinical characteristics of coronavirus disease 2019 in China. N Engl J Med. 2020;382(18):1708‐1720. 32109013 10.1056/NEJMoa2002032 PMC7092819

[25] Huang C , Wang Y , Li X , et al. Clinical features of patients infected with 2019 novel coronavirus in Wuhan, China. Lancet. 2020;395(10223):497‐506. 31986264 10.1016/S0140-6736(20)30183-5 PMC7159299

[26] Andersen CL , Tesfa D , Siersma VD , et al. Prevalence and clinical significance of neutropenia discovered in routine complete blood cell counts: a longitudinal study. J Intern Med. 2016;279(6):566‐575. 26791682 10.1111/joim.12467

[27] Williamson DR , Albert M , Heels‐Ansdell D , et al. Thrombocytopenia in critically ill patients receiving thromboprophylaxis: frequency, risk factors, and outcomes. Chest. 2013;144(4):1207‐1215. 23788287 10.1378/chest.13-0121

[28] U.S. Department of Health and Human Services NIH National Cancer Institute Common terminology criteria for adverse events (CTCAE) Version 4.0. 2010. Accessed June 6, 2016. http://evs.nci.nih.gov/ftp1/CTCAE/CTCAE_4.03_2010-06-14_QuickReference_5x7.pdf

[29] Farrington PWH , Ghebremichael‐Weldeselassie Y . Self‐controlled case series studies: A modelling guide with R. Chapman & Hall/CRC Press; 2018.

[30] Black C , Kaye JA , Jick H . MMR vaccine and idiopathic thrombocytopaenic purpura. Br J Clin Pharmacol. 2003;55(1):107‐111. 12534647 10.1046/j.1365-2125.2003.01790.x PMC1884189

[31] Sing CW , Wong IC , Cheung BM , Chan JC , Chu JK , Cheung CL . Incidence and risk estimate of drug‐induced agranulocytosis in Hong Kong Chinese. A population‐based case‐control study. Pharmacoepidemiol Drug Saf. 2017;26(3):248‐255. 28083886 10.1002/pds.4156

[32] Schultz NH , Sørvoll IH , Michelsen AE , et al. Thrombosis and thrombocytopenia after ChAdOx1 nCoV‐19 vaccination. N Engl J Med. 2021;384(22):2124‐2130. 33835768 10.1056/NEJMoa2104882 PMC8112568

[33] Klein NP , Lewis N , Goddard K , et al. Surveillance for adverse events after COVID‐19 mRNA vaccination. Jama. 2021;326:1390‐1399. 34477808 10.1001/jama.2021.15072 PMC8511971

[34] Cummins D , Wilson ME , Foulger KJ , Dawson D , Hogarth AM . Haematological changes associated with influenza vaccination in people aged over 65: case report and prospective study. Clin Lab Haematol. 1998;20(5):285‐287. 9807675 10.1046/j.1365-2257.1998.00149.x

[35] Griffin M , Makris M . Vaccination induced neutropenia. Int J Lab Hematol. 2013;35(5):e33. 23521785 10.1111/ijlh.12063

[36] Lim WW , Mak L , Leung GM , Cowling BJ , Peiris M . Comparative immunogenicity of mRNA and inactivated vaccines against COVID‐19. Lancet Microbe. 2021;2:e423. 34308395 10.1016/S2666-5247(21)00177-4 PMC8282488

[37] Mulligan MJ , Lyke KE , Kitchin N , et al. Phase I/II study of COVID‐19 RNA vaccine BNT162b1 in adults. Nature. 2020;586(7830):589‐593. 32785213 10.1038/s41586-020-2639-4

[38] Acharya S , Bussel JB . Hematologic toxicity of sodium valproate. J Pediatr Hematol Oncol. 2000;22(1):62‐65. 10695824 10.1097/00043426-200001000-00012

[39] Nongpiur A , Praharaj SK , Sarkar S , Das B . Delayed onset of clozapine‐induced leucopenia. Am J Ther. 2012;19(3):e118‐e119. 20724912 10.1097/MJT.0b013e3181ebb268

[40] Atallah‐Yunes SA , Ready A , Newburger PE . Benign ethnic neutropenia. Blood Rev. 2019;37:100586. 31255364 10.1016/j.blre.2019.06.003 PMC6702066

[41] Census and Statistics Department . Hong Kong 2016 Population By‐census—Thematic Report: Ethnic Minorities. Hong Kong Special Administrative Region; 2017.

