# Supplementary material for: COVID‐19 vaccines and risks of hematological abnormalities: Nested case–control and self‐controlled case series study
Source: Am J Hematol. 2022 Feb 9;97(4):470–80. doi: 10.1002/ajh.26478 (PMC9011752; doi:10.1002/ajh.26478)
Supplement: Supplementary file 1 — Table S1. Rollout schedule for the COVID‐19 Vaccination Programme in Hong Kong. Table S2. Definition for diagnosis, procedure and prescription. Table S3. Incidence of thrombocytopenia and neutropenia stratified by disease severity after COVID‐19 vaccine. Table S4. Subgroup analysis in people aged <60 in nested case‐control analysis. Table S5. Subgroup analysis in people aged ≥60 in nested case‐control analysis. Table S6. Subgroup analysis in people aged <60 in SCCS analysis. Table S7. Subgroup analysis in people aged ≥60 in SCCS analysis. Table S8. Sensitivity analysis excluding people with SARS‐CoV‐2 positive test in nested case‐control analysis. Table S9. Sensitivity analysis excluding people with SARS‐CoV‐2 positive test in SCCS analysis. Table S10. Sensitivity analysis using a longer timeframe (84 days) to define hematological abnormalities associated with COVID‐19 vaccination in nested case‐control analysis. Table S11. Sensitivity analysis using a longer timeframe (84 days) to define hematological abnormalities associated with COVID‐19 vaccination in SCCS analysis. Figure S1. Schema for SCCS design. Figure S2. Screening flow chart for nested case‐control and SCCS analysis. Figure S3. Time between second dose vaccination and outcome occurrence. [file AJH-97--s001.docx]

**Title: COVID-19 vaccines and risks of hematological abnormalities: nested case-control and self-controlled case series study.**

**Supplementary data**

Supplementary Table 1 Rollout schedule for the COVID-19 Vaccination Programme in Hong Kong

Supplementary Table 2 Definition for diagnosis, procedure and prescription

Supplementary Table 3 Incidence of thrombocytopenia and neutropenia stratified by disease severity after COVID-19 vaccine

Supplementary Table 4 Subgroup analysis in people aged <60 in nested case-control analysis

Supplementary Table 5 Subgroup analysis in people aged ≥60 in nested case-control analysis

Supplementary Table 6 Subgroup analysis in people aged <60 in SCCS analysis

Supplementary Table 7 Subgroup analysis in people aged ≥60 in SCCS analysis

Supplementary Table 8 Sensitivity analysis excluding people with SARS-COV-2 positive test in nested case-control analysis

Supplementary Table 9 Sensitivity analysis excluding people with SARS-COV-2 positive test in SCCS analysis

Supplementary Table 10 Sensitivity analysis using a longer timeframe (84 days) to define hematological abnormalities associated with COVID-19 vaccination in nested case-control analysis

Supplementary Table 11 Sensitivity analysis using a longer timeframe (84 days) to define hematological abnormalities associated with COVID-19 vaccination in SCCS analysis

Supplementary Figure 1 Schema for SCCS design

Supplementary Figure 2 Screening flow chart for nested case-control and SCCS analysis

Supplementary Figure 3 Time between second dose vaccination and outcome occurrence

**Supplementary Table 1 Rollout schedule for the COVID-19 Vaccination Programme in Hong Kong**

| Phase | Date of rollout | Eligibility for vaccination |
| --- | --- | --- |
| 1 [1] | 26 February 2021 | - Healthcare staff and staff involved in anti-epidemic work - Persons aged 60 or above and a maximum of two carers accompanying elderly people aged above 70 - Residents and staff of residential care homes for the elderly and persons with disabilities - People providing essential public services - People providing cross-boundary transportation or working at control points and ports |
| 2[2] | 8 March 2021 | - Staff of food and beverages premises, markets, supermarkets, convenience stores, couriers and takeaway delivery - Staff of local public transport service operators - Registered construction workers - Staff of property management - Teachers and school staff - Staff in the tourism industry - Staff of scheduled premises under the Prevention and Control of Disease |
| 3 [3] | 16 March 2021 | - People aged between 30 and 59 - Students aged 16 or above studying outside Hong Kong - Domestic helpers |
| 4 [4] | 15 April 2021 | - People aged 16 to 29 (18 for person receiving CoronaVac) |
| 5 [5] | 14 June 2021 | - People aged 12 to 15 for BNT162b2 |

References:

1. The Government of the Hong Kong Special Administrative Region. Government announces 2019 COVID-19 Vaccination Programme. Press Releases. 18 Feb 2021 (https://www.info.gov.hk/gia/general/202102/18/P2021021800767.htm)

2. The Government of the Hong Kong Special Administrative Region. Government expands scope of priority groups and opens more CVCs. Press Releases. 8 Mar 2021 (https://www.info.gov.hk/gia/general/202103/08/P2021030800738.htm)

3. The Government of the Hong Kong Special Administrative Region. Vaccination priority groups to be expanded to cover people aged 30 or above. Press Releases. 15 Mar 2021 (https://www.info.gov.hk/gia/general/202103/15/P2021031500626.htm)

4. The Government of the Hong Kong Special Administrative Region. COVID-19 Vaccination Programme opens to persons aged 16 or above. Press Releases. 15 Apr 2021 (https://www.info.gov.hk/gia/general/202104/15/P2021041500565.htm)

5. The Government of the Hong Kong Special Administrative Region. COVID-19 Vaccination Programme opens to persons aged 16 or above. Press Releases. 3 Jun 2021 (https://www.info.gov.hk/gia/general/202106/03/P2021060300652.htm)

**Supplementary Table 2 Definition for diagnosis, procedure and prescription**

| Variable | Definition |
| --- | --- |
| **Haematological abnormality** | **Laboratory record** |
| Thrombocytopenia | Platelet counts <150 x10^9^/L |
| Leukopenia | White blood cell counts <4 x10^9^/L |
| Neutropenia | Neutrophil counts <1.5 x10^9^/L |
|  |  |
| **Medical diagnosis or procedure** | **ICD9** |
| Cancer | 140 – 208, V10 |
| Myelodysplastic syndromes | 238.7 |
| Iron deficiency anemia | 280 |
| Vitamin B12 deficiency anemia | 281.1, 281.2 |
| Aplastic anemia | 284 |
| Hypersplenism | 289.4 |
| Chemotherapy or radiotherapy | V58.0, V58.1 |
| Chronic renal failure | 585 |
| Viral infection | 042-059, 050-059, 066, 070-072, 074-075, 077-079, 138, 139.0 |
| Alcohol abuse / alcoholic liver disease | 303, 305.0, 571.1-571.3 |
|  |  |
| **Prescription** | **BNF** |
| Drugs for malignant disease and immunosuppression | 8.1 – 8.3 |
| Heparin | 2.8.1 with local drug code “HEPA” |

**Supplementary Table 3 Incidence of thrombocytopenia and neutropenia stratified by disease severity after COVID-19 vaccination**

|  | Case, n | | | Incidence (95% CI) |
| --- | --- | --- | --- | --- |
|  | After first dose and before second dose | After second | Total |  |
| **Thrombocytopenia**  **(Platelet counts, x10^9^/L)** |  |  |  |  |
| *CoronaVac* |  |  |  |  |
| Mild, <100 – 150 | 137 | 140 | 277 | 2.28 (2.02 - 2.56) |
| Moderate, 50 – 100 | 8 | 17 | 25 | 0.21 (0.13 - 0.30) |
| Severe, <50 | 1 | 3 | 4 | 0.03 (0.01 - 0.08) |
|  |  |  |  |  |
| *BNT162b2* |  |  |  |  |
| Mild, <100 – 150 | 70 | 109 | 179 | 1.20 (1.03 - 1.39) |
| Moderate, 50 – 100 | 9 | 12 | 21 | 0.14 (0.09 - 0.21) |
| Severe, <50 | 3 | 5 | 8 | 0.05 (0.02 - 0.11) |
| **Neutropenia**  **(Neutrophil counts, x10^9^/L)** |  |  |  |  |
| *CoronaVac* |  |  |  |  |
| Mild, <1.0 – 1.5 | 7 | 8 | 15 | 0.12 (0.07 - 0.20) |
| Moderate, 0.5 – 1.0 | 2 | 0 | 2 | 0.02 (0.00 - 0.06) |
| Severe, <0.5 | 0 | 2 | 2 | 0.02 (0.00 - 0.06) |
|  |  |  |  |  |
| *BNT162b2* |  |  |  |  |
| Mild, <1.0 – 1.5 | 9 | 21 | 30 | 0.20 (0.13 - 0.28) |
| Moderate, 0.5 – 1.0 | 0 | 10 | 10 | 0.07 (0.03 - 0.12) |
| Severe, <0.5 | 0 | 0 | 0 | 0 |

**Supplementary Table 4 Subgroup analysis in people aged <60 in nested case-control analysis**

| Exposure | Case | Control | OR  (95% CI) | Adjusted* OR  (95% CI) |
| --- | --- | --- | --- | --- |
| **Thrombocytopenia** |  |  |  |  |
| *Events after first dose and before second dose* |  |  |  |  |
| Not vaccinated | 1668 | 14198 | 1 | 1 |
| CoronaVac | 69 | 652 | 0.88 (0.69 - 1.14) | 0.92 (0.71 - 1.19) |
| BNT162b2 | 69 | 826 | 0.70 (0.54 - 0.9) | 0.71 (0.55 - 0.91) |
| *Events after second dose* |  |  |  |  |
| Not vaccinated | 1668 | 13931 | 1 | 1 |
| CoronaVac | 41 | 447 | 0.73 (0.53 - 1.01) | 0.73 (0.52 - 1.01) |
| BNT162b2 | 66 | 672 | 0.80 (0.62 - 1.04) | 0.79 (0.61 - 1.03) |
| **Leukopenia** |  |  |  |  |
| *Events after first dose and before second dose* |  |  |  |  |
| Not vaccinated | 895 | 7743 | 1 | 1 |
| CoronaVac | 37 | 354 | 0.88 (0.62 - 1.25) | 0.89 (0.63 - 1.26) |
| BNT162b2 | 54 | 464 | 0.97 (0.73 - 1.30) | 0.96 (0.72 - 1.29) |
| *Events after second dose* |  |  |  |  |
| Not vaccinated | 895 | 7765 | 1 | 1 |
| CoronaVac | 25 | 265 | 0.79 (0.52 - 1.20) | 0.77 (0.5 - 1.17) |
| BNT162b2 | 68 | 368 | 1.56 (1.19 - 2.05) | 1.58 (1.21 - 2.08) |
| **Neutropenia** |  |  |  |  |
| *Events after first dose and before second dose* |  |  |  |  |
| Not vaccinated | 176 | 1600 | 1 | 1 |
| CoronaVac | 7 | 65 | 0.94 (0.42 - 2.09) | 0.81 (0.35 - 1.87) |
| BNT162b2 | 15 | 105 | 1.23 (0.69 - 2.19) | 1.25 (0.70 - 2.26) |
| *Events after second dose* |  |  |  |  |
| Not vaccinated | 176 | 1602 | 1 | 1 |
| CoronaVac | 4 | 37 | 0.92 (0.32 - 2.63) | 0.87 (0.30 - 2.52) |
| BNT162b2 | 17 | 54 | 2.64 (1.50 - 4.67) | 2.76 (1.54 - 4.94) |

OR odd ratio

*model adjusted for medical history of diabetes, hypertension, rheumatoid arthritis, systemic lupus erythematosus, psoriasis, thyroid disorders, moderate/server liver diseases; recent (90 days prior) prescription of lipid-lowering agents, antiepileptic drugs, diuretics, oral anticoagulants, non-steroidal anti-inflammatory drugs, antithyroid drugs, antipsychotic drugs, antiplatelet drugs, antiarrthymic drugs

**Supplementary Table 5 Subgroup analysis in people aged ≥60 in nested case-control analysis**

| Exposure | Case | Control | OR  (95% CI) | Adjusted* OR  (95% CI) |
| --- | --- | --- | --- | --- |
| **Thrombocytopenia** |  |  |  |  |
| *Events after first dose and before second dose* |  |  |  |  |
| Not vaccinated | 3391 | 31726 | 1 | 1 |
| CoronaVac | 97 | 725 | 1.05 (0.89 - 1.24) | 1.22 (0.98 - 1.52) |
| BNT162b2 | 38 | 377 | 0.76 (0.62 - 0.93) | 0.86 (0.61 - 1.20) |
| *Events after second dose* |  |  |  |  |
| Not vaccinated | 3391 | 31404 | 1 | 1 |
| CoronaVac | 66 | 572 | 0.89 (0.72 - 1.09) | 1.01 (0.78 - 1.31) |
| BNT162b2 | 33 | 295 | 0.86 (0.70 - 1.06) | 0.98 (0.68 - 1.41) |
| **Leukopenia** |  |  |  |  |
| *Events after first dose and before second dose* |  |  |  |  |
| Not vaccinated | 794 | 7381 | 1 | 1 |
| CoronaVac | 31 | 225 | 1.02 (0.79 - 1.32) | 1.22 (0.83 - 1.79) |
| BNT162b2 | 11 | 111 | 0.96 (0.74 - 1.25) | 0.94 (0.50 - 1.76) |
| *Events after second dose* |  |  |  |  |
| Not vaccinated | 794 | 7302 | 1 | 1 |
| CoronaVac | 17 | 143 | 0.89 (0.64 - 1.22) | 1.09 (0.65 - 1.81) |
| BNT162b2 | 15 | 91 | 1.56 (1.22 - 1.98) | 1.58 (0.90 - 2.77) |
| **Neutropenia** |  |  |  |  |
| *Events after first dose and before second dose* |  |  |  |  |
| Not vaccinated | 121 | 1078 | 1 | 1 |
| CoronaVac | 2 | 34 | 0.80 (0.40 - 1.60) | 0.40 (0.08 - 1.95) |
| BNT162b2 | 1 | 18 | 1.11 (0.64 - 1.92) | 0.48 (0.06 - 3.73) |
| *Events after second dose* |  |  |  |  |
| Not vaccinated | 121 | 1100 | 1 | 1 |
| CoronaVac | 2 | 28 | 0.81 (0.35 - 1.88) | 0.68 (0.16 - 2.91) |
| BNT162b2 | 4 | 14 | 2.63 (1.58 - 4.38) | 2.54 (0.79 - 8.16) |

OR odd ratio

*model adjusted for medical history of diabetes, hypertension, rheumatoid arthritis, systemic lupus erythematosus, psoriasis, thyroid disorders, moderate/server liver diseases; recent (90 days prior) prescription of lipid-lowering agents, antiepileptic drugs, diuretics, oral anticoagulants, non-steroidal anti-inflammatory drugs, antithyroid drugs, antipsychotic drugs, antiplatelet drugs, antiarrthymic drugs

**Supplementary Table 6 Subgroup analysis in people aged <60 in SCCS analysis**

| Risk Period | Event | Person-years | IR | IRR* (95% CI) |
| --- | --- | --- | --- | --- |
| **Thrombocytopenia** |  |  |  |  |
| CoronaVac |  |  |  |  |
| Control period | 2604 | 1139.32 | 2.29 |  |
| 1st dose, day 0 to 13 | 39 | 17.06 | 2.29 | 0.88 (0.58-1.35) |
| 1st dose, day 14 to 27 | 51 | 16.45 | 3.10 | 1.15 (0.78-1.71) |
| 2nd dose, day 0 to 13 | 30 | 11.89 | 2.52 | 0.91 (0.54-1.54) |
| 2nd dose, day 14 to 27 | 21 | 10.23 | 2.05 | 0.68 (0.39-1.19) |
|  |  |  |  |  |
| BNT162b2 |  |  |  |  |
| Control period | 2693 | 1182.27 | 2.28 |  |
| 1st dose, day 0 to 13 | 49 | 20.71 | 2.37 | 0.97 (0.67-1.40) |
| 1st dose, day 14 to 27 | 34 | 13.40 | 2.54 | 0.95 (0.64-1.41) |
| 2nd dose, day 0 to 13 | 54 | 13.31 | 4.06 | 1.47 (1.02-2.11) |
| 2nd dose, day 14 to 27 | 21 | 11.40 | 1.84 | 0.63 (0.38-1.04) |
| **Leukopenia** |  |  |  |  |
| CoronaVac |  |  |  |  |
| Control period | 1393 | 611.32 | 2.28 |  |
| 1st dose, day 0 to 13 | 23 | 10.48 | 2.19 | 0.84 (0.48-1.46) |
| 1st dose, day 14 to 27 | 27 | 9.78 | 2.76 | 0.90 (0.55-1.49) |
| 2nd dose, day 0 to 13 | 21 | 7.01 | 3.00 | 1.06 (0.62-1.83) |
| 2nd dose, day 14 to 27 | 17 | 6.12 | 2.78 | 1.05 (0.59-1.87) |
|  |  |  |  |  |
| BNT162b2 |  |  |  |  |
| Control period | 1488 | 670.81 | 2.22 |  |
| 1st dose, day 0 to 13 | 49 | 16.09 | 3.05 | 1.29 (0.86-1.92) |
| 1st dose, day 14 to 27 | 25 | 10.73 | 2.33 | 0.94 (0.59-1.51) |
| 2nd dose, day 0 to 13 | 69 | 11.06 | 6.24 | 2.61 (1.76-3.86) |
| 2nd dose, day 14 to 27 | 19 | 9.58 | 1.98 | 0.77 (0.44-1.33) |
| **Neutropenia** |  |  |  |  |
| CoronaVac |  |  |  |  |
| Control period | 325 | 142.42 | 2.28 |  |
| 1st dose, day 0 to 13 | 7 | 2.00 | 3.50 | 1.35 (0.53-3.41) |
| 1st dose, day 14 to 27 | 4 | 1.94 | 2.06 | 0.83 (0.30-2.28) |
| 2nd dose, day 0 to 13 | 4 | 1.29 | 3.10 | 1.29 (0.44-3.80) |
| 2nd dose, day 14 to 27 | 2 | 1.23 | 1.63 | 0.66 (0.13-3.45) |
|  |  |  |  |  |
| BNT162b2 |  |  |  |  |
| Control period | 372 | 165.95 | 2.24 |  |
| 1st dose, day 0 to 13 | 11 | 4.37 | 2.52 | 0.38 (0.12-1.26) |
| 1st dose, day 14 to 27 | 5 | 2.92 | 1.71 | 0.24 (0.07-0.83) |
| 2nd dose, day 0 to 13 | 17 | 3.03 | 5.61 | 1.09 (0.44-2.68) |
| 2nd dose, day 14 to 27 | 6 | 2.65 | 2.26 | 0.49 (0.17-1.40) |

IR incidence rate; IRR incidence rate ratio

*IRR were estimated using modified SCCS extension “eventdepenexp” model

**Supplementary Table 7 Subgroup analysis in people aged ≥60 in SCCS analysis**

| Risk Period | Event | Person-years | IR | IRR* (95% CI) |
| --- | --- | --- | --- | --- |
| **Thrombocytopenia** |  |  |  |  |
| CoronaVac |  |  |  |  |
| Control period | 6767 | 2969.86 | 2.28 |  |
| 1st dose, day 0 to 13 | 64 | 22.32 | 2.87 | 1.13 (0.82-1.55) |
| 1st dose, day 14 to 27 | 65 | 21.25 | 3.06 | 1.17 (0.86-1.60) |
| 2nd dose, day 0 to 13 | 50 | 15.60 | 3.21 | 1.19 (0.83-1.72) |
| 2nd dose, day 14 to 27 | 46 | 14.70 | 3.13 | 1.09 (0.76-1.57) |
|  |  |  |  |  |
| BNT162b2 |  |  |  |  |
| Control period | 6594 | 2877.74 | 2.29 |  |
| 1st dose, day 0 to 13 | 31 | 12.03 | 2.58 | 0.99 (0.63-1.55) |
| 1st dose, day 14 to 27 | 25 | 8.22 | 3.04 | 1.11 (0.69-1.76) |
| 2nd dose, day 0 to 13 | 21 | 8.76 | 2.40 | 0.84 (0.50-1.41) |
| 2nd dose, day 14 to 27 | 24 | 7.71 | 3.11 | 1.05 (0.65-1.68) |
| **Leukopenia** |  |  |  |  |
| CoronaVac |  |  |  |  |
| Control period | 2394 | 1046.96 | 2.29 |  |
| 1st dose, day 0 to 13 | 27 | 8.01 | 3.37 | 1.32 (0.80-2.18) |
| 1st dose, day 14 to 27 | 19 | 7.48 | 2.54 | 0.95 (0.54-1.69) |
| 2nd dose, day 0 to 13 | 12 | 5.40 | 2.22 | 0.87 (0.43-1.79) |
| 2nd dose, day 14 to 27 | 13 | 5.20 | 2.50 | 0.79 (0.40-1.56) |
|  |  |  |  |  |
| BNT162b2 |  |  |  |  |
| Control period | 2350 | 1027.94 | 2.29 |  |
| 1st dose, day 0 to 13 | 15 | 5.66 | 2.65 | 1.21 (0.62-2.34) |
| 1st dose, day 14 to 27 | 14 | 3.90 | 3.59 | 1.42 (0.78-2.58) |
| 2nd dose, day 0 to 13 | 12 | 4.02 | 2.99 | 1.36 (0.70-2.67) |
| 2nd dose, day 14 to 27 | 10 | 3.67 | 2.72 | 1.40 (0.66-2.94) |
| **Neutropenia** |  |  |  |  |
| CoronaVac |  |  |  |  |
| Control period | 431 | 187.14 | 2.30 |  |
| 1st dose, day 0 to 13 | 5 | 1.19 | 4.20 | 2.36 (0.70-7.93) |
| 1st dose, day 14 to 27 | 0 | 1.14 | 0.00 | 0.00 (0.00-0.00) |
| 2nd dose, day 0 to 13 | 1 | 0.88 | 1.14 | 0.51 (0.05-5.37) |
| 2nd dose, day 14 to 27 | 2 | 0.76 | 2.63 | 0.84 (0.19-3.81) |
|  |  |  |  |  |
| BNT162b2 |  |  |  |  |
| Control period | 429 | 186.21 | 2.30 |  |
| 1st dose, day 0 to 13 | 1 | 1.10 | 0.91 | 0.58 (0.10-3.58) |
| 1st dose, day 14 to 27 | 1 | 0.63 | 1.59 | 0.91 (0.12-6.73) |
| 2nd dose, day 0 to 13 | 2 | 0.78 | 2.56 | 1.32 (0.28-6.16) |
| 2nd dose, day 14 to 27 | 2 | 0.64 | 3.12 | 1.46 (0.28-7.67) |

IR incidence rate; IRR incidence rate ratio

*IRR were estimated using modified SCCS extension “eventdepenexp” model

**Supplementary Table 8 Sensitivity analysis excluding people with SARS-COV-2 positive test in nested case-control analysis**

| Exposure | Case | Control | OR  (95% CI) | Adjusted* OR  (95% CI) |
| --- | --- | --- | --- | --- |
| **Thrombocytopenia** |  |  |  |  |
| *Events after first dose and before second dose* |  |  |  |  |
| Not vaccinated | 5058 | 45860 | 1 | 1 |
| CoronaVac | 166 | 1412 | 1.02 (0.87 - 1.21) | 1.03 (0.87 - 1.22) |
| BNT162b2 | 106 | 1237 | 0.73 (0.60 - 0.90) | 0.73 (0.59 - 0.89) |
| *Events after second dose* |  |  |  |  |
| Not vaccinated | 5058 | 45308 | 1 | 1 |
| CoronaVac | 106 | 1013 | 0.89 (0.72 - 1.09) | 0.88 (0.72 - 1.08) |
| BNT162b2 | 98 | 991 | 0.83 (0.67 - 1.02) | 0.82 (0.66 - 1.01) |
| **Leukopenia** |  |  |  |  |
| *Events after first dose and before second dose* |  |  |  |  |
| Not vaccinated | 1688 | 15159 | 1 | 1 |
| CoronaVac | 68 | 583 | 1.02 (0.79 - 1.32) | 1.00 (0.77 - 1.30) |
| BNT162b2 | 65 | 597 | 0.93 (0.72 - 1.21) | 0.93 (0.72 - 1.21) |
| *Events after second dose* |  |  |  |  |
| Not vaccinated | 1688 | 15089 | 1 | 1 |
| CoronaVac | 41 | 393 | 0.90 (0.65 - 1.25) | 0.90 (0.64 - 1.25) |
| BNT162b2 | 83 | 462 | 1.55 (1.22 - 1.98) | 1.59 (1.25 - 2.03) |
| **Neutropenia** |  |  |  |  |
| *Events after first dose and before second dose* |  |  |  |  |
| Not vaccinated | 297 | 2681 | 1 | 1 |
| CoronaVac | 9 | 93 | 0.84 (0.42 - 1.69) | 0.76 (0.36 - 1.57) |
| BNT162b2 | 16 | 125 | 1.10 (0.64 - 1.90) | 1.13 (0.65 - 1.97) |
| *Events after second dose* |  |  |  |  |
| Not vaccinated | 297 | 2684 | 1 | 1 |
| CoronaVac | 5 | 64 | 0.67 (0.26 - 1.69) | 0.64 (0.25 - 1.65) |
| BNT162b2 | 21 | 82 | 2.2 (1.32 - 3.66) | 2.38 (1.42 - 3.98) |

OR odd ratio

*model adjusted for medical history of diabetes, hypertension, rheumatoid arthritis, systemic lupus erythematosus, psoriasis, thyroid disorders, moderate/server liver diseases; recent (90 days prior) prescription of lipid-lowering agents, antiepileptic drugs, diuretics, oral anticoagulants, non-steroidal anti-inflammatory drugs, antithyroid drugs, antipsychotic drugs, antiplatelet drugs, antiarrthymic drugs

**Supplementary Table 9 Sensitivity analysis excluding people with SARS-COV-2 positive test in SCCS analysis**

| Risk Period | Event | Person-years | IR | IRR* (95% CI) |
| --- | --- | --- | --- | --- |
| **Thrombocytopenia** |  |  |  |  |
| CoronaVac |  |  |  |  |
| Control period | 9367 | 4107.44 | 2.28 |  |
| 1st dose, day 0 to 13 | 103 | 39.38 | 2.62 | 1.04 (0.80-1.34) |
| 1st dose, day 14 to 27 | 116 | 37.70 | 3.08 | 1.18 (0.93-1.50) |
| 2nd dose, day 0 to 13 | 80 | 27.49 | 2.91 | 1.08 (0.80-1.46) |
| 2nd dose, day 14 to 27 | 67 | 24.94 | 2.69 | 0.92 (0.68-1.25) |
|  |  |  |  |  |
| BNT162b2 |  |  |  |  |
| Control period | 9282 | 4057.62 | 2.29 |  |
| 1st dose, day 0 to 13 | 80 | 32.67 | 2.45 | 0.96 (0.72-1.28) |
| 1st dose, day 14 to 27 | 58 | 21.54 | 2.69 | 0.98 (0.72-1.33) |
| 2nd dose, day 0 to 13 | 75 | 22.03 | 3.40 | 1.19 (0.89-1.59) |
| 2nd dose, day 14 to 27 | 45 | 19.07 | 2.36 | 0.79 (0.56-1.12) |
| **Leukopenia** |  |  |  |  |
| CoronaVac |  |  |  |  |
| Control period | 3787 | 1657.63 | 2.28 |  |
| 1st dose, day 0 to 13 | 50 | 18.42 | 2.71 | 1.00 (0.68-1.47) |
| 1st dose, day 14 to 27 | 44 | 17.18 | 2.56 | 0.85 (0.58-1.25) |
| 2nd dose, day 0 to 13 | 33 | 12.38 | 2.67 | 0.95 (0.61-1.46) |
| 2nd dose, day 14 to 27 | 30 | 11.29 | 2.66 | 0.90 (0.58-1.39) |
|  |  |  |  |  |
| BNT162b2 |  |  |  |  |
| Control period | 3835 | 1697.69 | 2.26 |  |
| 1st dose, day 0 to 13 | 64 | 21.66 | 2.95 | 1.23 (0.88-1.73) |
| 1st dose, day 14 to 27 | 39 | 14.56 | 2.68 | 1.06 (0.73-1.53) |
| 2nd dose, day 0 to 13 | 81 | 15.04 | 5.39 | 2.23 (1.60-3.10) |
| 2nd dose, day 14 to 27 | 29 | 13.21 | 2.20 | 0.91 (0.58-1.43) |
| **Neutropenia** |  |  |  |  |
| CoronaVac |  |  |  |  |
| Control period | 755 | 329.13 | 2.29 |  |
| 1st dose, day 0 to 13 | 12 | 3.19 | 3.76 | 1.31 (0.59-2.90) |
| 1st dose, day 14 to 27 | 4 | 3.07 | 1.30 | 0.50 (0.18-1.38) |
| 2nd dose, day 0 to 13 | 5 | 2.17 | 2.30 | 0.92 (0.33-2.52) |
| 2nd dose, day 14 to 27 | 4 | 1.99 | 2.01 | 0.69 (0.22-2.15) |
|  |  |  |  |  |
| BNT162b2 |  |  |  |  |
| Control period | 800 | 351.72 | 2.27 |  |
| 1st dose, day 0 to 13 | 12 | 5.47 | 2.19 | 0.41 (0.15-1.09) |
| 1st dose, day 14 to 27 | 6 | 3.55 | 1.69 | 0.28 (0.10-0.85) |
| 2nd dose, day 0 to 13 | 19 | 3.81 | 4.99 | 1.10 (0.52-2.31) |
| 2nd dose, day 14 to 27 | 8 | 3.29 | 2.43 | 0.60 (0.25-1.46) |

IR incidence rate; IRR incidence rate ratio

*IRR were estimated using modified SCCS extension “eventdepenexp” model

Supplementary Table 10 Sensitivity analysis using a longer timeframe (84 days) to define hematological abnormalities associated with COVID-19 vaccination in nested case-control analysis

| Exposure | Case | Control | OR  (95% CI) | Adjusted* OR  (95% CI) |
| --- | --- | --- | --- | --- |
| **Thrombocytopenia** |  |  |  |  |
| *Events after first dose and before second dose* |  |  |  |  |
| Not vaccinated | 5059 | 45924 | 1 | 1 |
| CoronaVac | 166 | 1377 | 1.05 (0.89 - 1.24) | 1.07 (0.90 - 1.26) |
| BNT162b2 | 107 | 1203 | 0.76 (0.62 - 0.93) | 0.75 (0.62 - 0.92) |
| *Events after second dose* |  |  |  |  |
| Not vaccinated | 5059 | 47305 | 1 | 1 |
| CoronaVac | 243 | 2223 | 0.99 (0.86 - 1.14) | 0.98 (0.85 - 1.12) |
| BNT162b2 | 205 | 1979 | 0.93 (0.80 - 1.08) | 0.93 (0.80 - 1.08) |
| **Leukopenia** |  |  |  |  |
| *Events after first dose and before second dose* |  |  |  |  |
| Not vaccinated | 1689 | 15124 | 1 | 1 |
| CoronaVac | 68 | 579 | 1.02 (0.79 - 1.32) | 1.01 (0.78 - 1.31) |
| BNT162b2 | 65 | 575 | 0.96 (0.74 - 1.25) | 0.96 (0.74 - 1.25) |
| *Events after second dose* |  |  |  |  |
| Not vaccinated | 1689 | 16014 | 1 | 1 |
| CoronaVac | 105 | 858 | 1.15 (0.93 - 1.42) | 1.15 (0.93 - 1.42) |
| BNT162b2 | 141 | 920 | 1.43 (1.18 - 1.72) | 1.46 (1.21 - 1.76) |
| **Neutropenia** |  |  |  |  |
| *Events after first dose and before second dose* |  |  |  |  |
| Not vaccinated | 297 | 2678 | 1 | 1 |
| CoronaVac | 9 | 99 | 0.8 (0.40 - 1.60) | 0.73 (0.35 - 1.49) |
| BNT162b2 | 16 | 123 | 1.11 (0.64 - 1.92) | 1.13 (0.65 - 1.98) |
| *Events after second dose* |  |  |  |  |
| Not vaccinated | 297 | 2918 | 1 | 1 |
| CoronaVac | 19 | 146 | 1.29 (0.78 - 2.13) | 1.29 (0.78 - 2.16) |
| BNT162b2 | 38 | 167 | 2.32 (1.56 - 3.45) | 2.38 (1.59 - 3.58) |

OR odd ratio

*model adjusted for medical history of diabetes, hypertension, rheumatoid arthritis, systemic lupus erythematosus, psoriasis, thyroid disorders, moderate/server liver diseases; recent (90 days prior) prescription of lipid-lowering agents, antiepileptic drugs, diuretics, oral anticoagulants, non-steroidal anti-inflammatory drugs, antithyroid drugs, antipsychotic drugs, antiplatelet drugs, antiarrthymic drugs

Supplementary Table 11 Sensitivity analysis using a longer timeframe (84 days) to define hematological abnormalities associated with COVID-19 vaccination in SCCS analysis

| Risk Period | Event | Person-years | IR | IRR* (95% CI) |
| --- | --- | --- | --- | --- |
| **Thrombocytopenia** |  |  |  |  |
| CoronaVac |  |  |  |  |
| Control period | 9182 | 4037.74 | 2.27 |  |
| 1st dose, day 0 to 13 | 103 | 39.38 | 2.62 | 1.06 (0.78-1.44) |
| 1st dose, day 14 to 27 | 116 | 37.70 | 3.08 | 1.20 (0.90-1.62) |
| 2nd dose, day 0 to 13 | 80 | 27.49 | 2.91 | 1.11 (0.77-1.59) |
| 2nd dose, day 14 to 27 | 67 | 24.94 | 2.69 | 0.94 (0.66-1.36) |
| 2nd dose, day 28 to 55 | 99 | 39.42 | 2.51 | 0.98 (0.72-1.33) |
| 2nd dose, day 56 to 83 | 90 | 32.02 | 2.81 | 1.12 (0.81-1.53) |
|  |  |  |  |  |
| BNT162b2 |  |  |  |  |
| Control period | 9141 | 4011.22 | 2.28 |  |
| 1st dose, day 0 to 13 | 80 | 32.74 | 2.44 | 0.74 (0.48-1.15) |
| 1st dose, day 14 to 27 | 59 | 21.62 | 2.73 | 0.78 (0.51-1.20) |
| 2nd dose, day 0 to 13 | 75 | 22.07 | 3.40 | 0.91 (0.58-1.43) |
| 2nd dose, day 14 to 27 | 45 | 19.11 | 2.35 | 0.60 (0.36-0.99) |
| 2nd dose, day 28 to 55 | 76 | 28.76 | 2.64 | 0.63 (0.41-0.99) |
| 2nd dose, day 56 to 83 | 70 | 20.03 | 3.49 | 0.99 (0.63-1.56) |
| **Leukopenia** |  |  |  |  |
| CoronaVac |  |  |  |  |
| Control period | 3692 | 1626.80 | 2.27 |  |
| 1st dose, day 0 to 13 | 50 | 18.49 | 2.70 | 1.56 (0.93-2.63) |
| 1st dose, day 14 to 27 | 46 | 17.26 | 2.67 | 1.37 (0.82-2.29) |
| 2nd dose, day 0 to 13 | 33 | 12.42 | 2.66 | 1.57 (0.85-2.89) |
| 2nd dose, day 14 to 27 | 30 | 11.33 | 2.65 | 1.49 (0.80-2.75) |
| 2nd dose, day 28 to 55 | 63 | 17.55 | 3.59 | 2.15 (1.25-3.71) |
| 2nd dose, day 56 to 83 | 32 | 13.92 | 2.30 | 1.34 (0.73-2.48) |
|  |  |  |  |  |
| BNT162b2 |  |  |  |  |
| Control period | 3756 | 1666.63 | 2.25 |  |
| 1st dose, day 0 to 13 | 64 | 21.75 | 2.94 | 1.34 (0.74-2.42) |
| 1st dose, day 14 to 27 | 39 | 14.64 | 2.66 | 1.14 (0.63-2.06) |
| 2nd dose, day 0 to 13 | 81 | 15.08 | 5.37 | 2.43 (1.28-4.62) |
| 2nd dose, day 14 to 27 | 29 | 13.25 | 2.19 | 1.00 (0.49-2.06) |
| 2nd dose, day 28 to 55 | 58 | 19.64 | 2.95 | 1.25 (0.64-2.44) |
| 2nd dose, day 56 to 83 | 24 | 12.48 | 1.92 | 0.80 (0.38-1.68) |
| **Neutropenia** |  |  |  |  |
| CoronaVac |  |  |  |  |
| Control period | 737 | 324.22 | 2.27 |  |
| 1st dose, day 0 to 13 | 12 | 3.19 | 3.76 | 2.55 (0.71-9.20) |
| 1st dose, day 14 to 27 | 4 | 3.07 | 1.30 | 0.90 (0.24-3.39) |
| 2nd dose, day 0 to 13 | 5 | 2.17 | 2.30 | 2.07 (0.38-11.20) |
| 2nd dose, day 14 to 27 | 4 | 1.99 | 2.01 | 1.62 (0.26-10.28) |
| 2nd dose, day 28 to 55 | 10 | 3.02 | 3.31 | 2.38 (0.50-11.31) |
| 2nd dose, day 56 to 83 | 9 | 2.32 | 3.88 | 3.10 (0.55-17.37) |
|  |  |  |  |  |
| BNT162b2 |  |  |  |  |
| Control period | 774 | 344.32 | 2.25 |  |
| 1st dose, day 0 to 13 | 12 | 5.47 | 2.19 | 1.18 (0.22-6.44) |
| 1st dose, day 14 to 27 | 6 | 3.55 | 1.69 | 0.78 (0.13-4.62) |
| 2nd dose, day 0 to 13 | 19 | 3.81 | 4.99 | 3.39 (0.57-20.29) |
| 2nd dose, day 14 to 27 | 8 | 3.29 | 2.43 | 1.79 (0.30-10.83) |
| 2nd dose, day 28 to 55 | 20 | 4.91 | 4.07 | 3.92 (0.67-23.13) |
| 2nd dose, day 56 to 83 | 7 | 2.93 | 2.39 | 2.24 (0.32-15.52) |

IR incidence rate; IRR incidence rate ratio

*IRR were estimated using modified SCCS extension “eventdepenexp” model


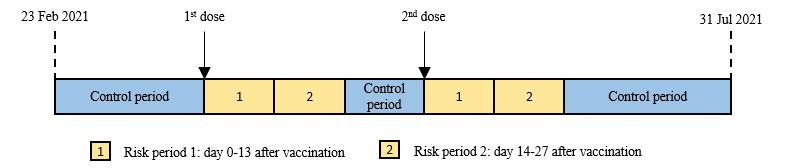


Supplementary Figure 1 Schema for SCCS design


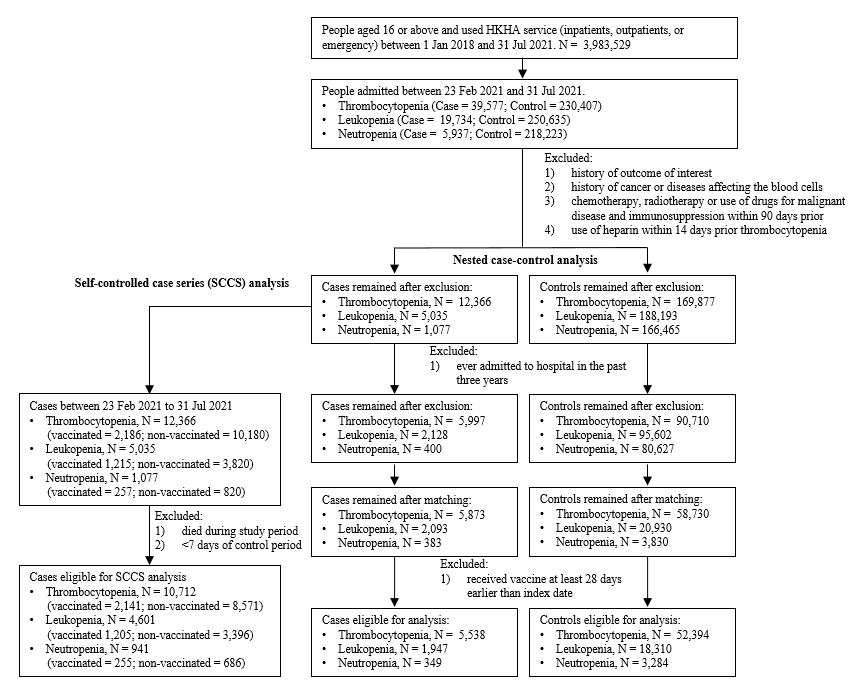


Supplementary Figure 2 Screening flow chart for nested case-control and SCCS analysis


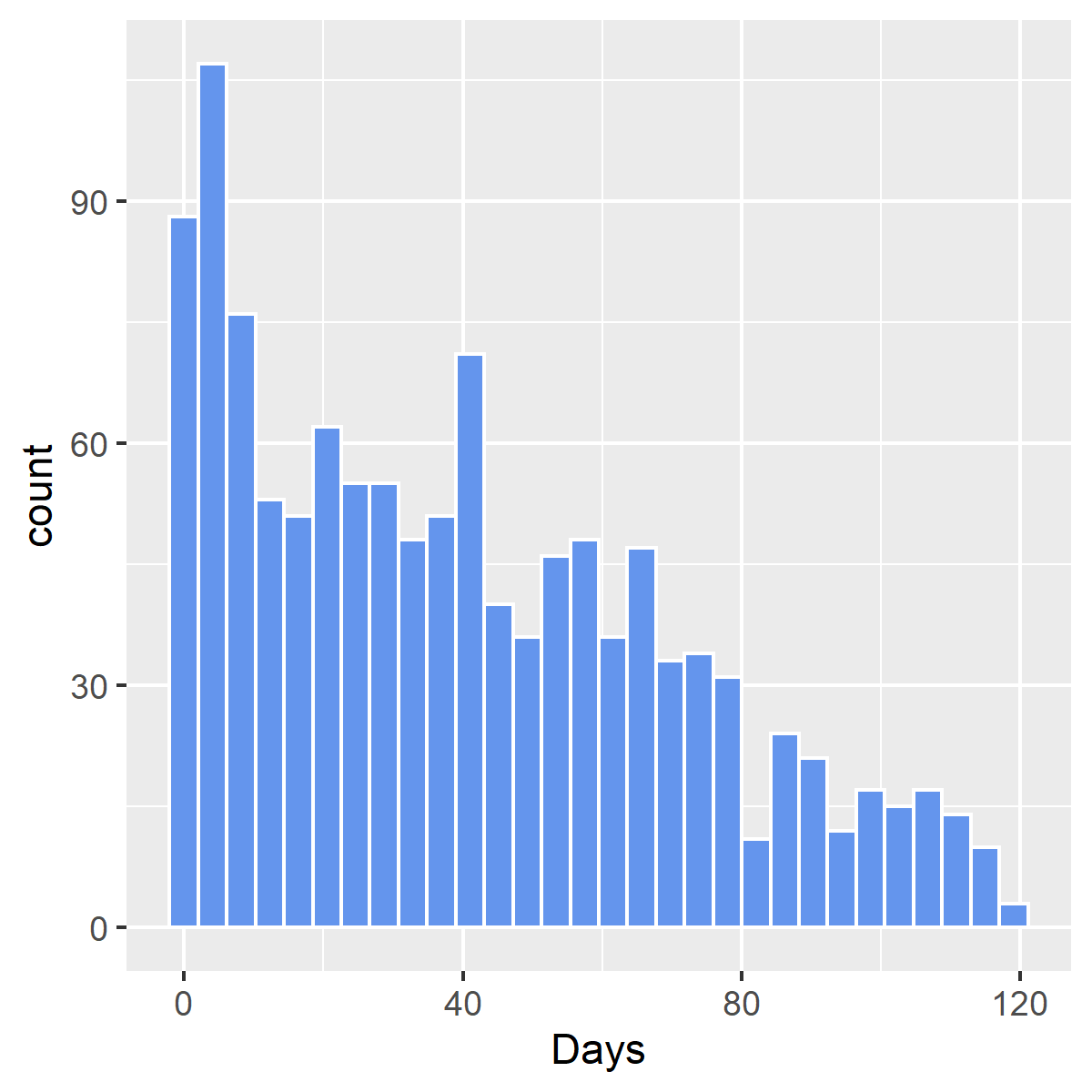


Supplementary Figure 3 Time between second dose vaccination and outcome occurrence
